# The reliability of the health related quality of life questionnaire PedsQL 3.0 cancer module in a sample of Swedish children

**DOI:** 10.1186/s12887-020-02387-0

**Published:** 2020-10-28

**Authors:** Peter Sand, Anna Nilsson Kleiberg, Marizela Kljajić, Birgitta Lannering

**Affiliations:** 1 Department of Psychiatry and Neurochemistry, Institute of Neuroscience and Physiology, Sahlgrenska Academy, Gothenburg University, Sahlgrenska University Hospital, Gothenburg, Sweden; 2 The Queen Silvia Children’s Hospital Sahlgrenska University Hospital , Gothenburg, Sweden; 3 Department of Plastic Surgery, Institute of Clinical Sciences, Sahlgrenska Academy, Sahlgrenska University Hospital, Gothenburg University, Gothenburg, Sweden; 4 Department of Pediatrics, Institute of Clinical Sciences, Sahlgrenska Academy, Gothenburg University, Gothenburg, Sweden

**Keywords:** Children, Parents, Cancer, Health-related quality of life

## Abstract

**Background:**

The Pediatric Quality of Life Inventory (PedsQL) is a modular instrument, designed to integrate generic and disease specific measures, and includes both self- and proxy-reports. The aim of the study was to assess the reliability and limited validity of the Swedish version of the disease specific Pediatric Quality of Life Inventory 3.0 Cancer Module Scales (PedsQL 3.0), in a sample of Swedish children diagnosed with cancer.

**Method:**

A total of 94 families at The Queen Silvia Children’s Hospital, Sahlgrenska University participated in the study. The Pediatric Quality of Life Inventory 4.0 Generic Core Scales (PedsQL 4.0) and the PedsQL 3.0 were administered to 63 children (aged 5–18 years) with cancer and to 94 parents of children with cancer aged 2–18 years.

**Results:**

The internal consistency of the PedsQL 3.0, reached or exceeded Cronbach’s alpha values of 0.70 for both -self- and proxy-reports. The PedsQL 4.0 and PedsQL 3.0 were highly correlated (*r* = 0.94 for proxy-reports and r = 0.91 for self-reports), indicating convergent validity.

**Conclusion:**

PedsQL 3.0 Cancer Module Scales can be used as a valuable tool for measuring cancer-specific HRQOL in child populations, both in research and in clinical practice.

## Background

The advances in cancer treatment over the past decades have increased the survival rate in childhood cancer. As of today four out of five children survive their cancer diagnosis, however with the risk of physical, neurocognitive and psychosocial long term side effects. The psychosocial consequences of pediatric cancer are well documented, as are the needs for routinely systematic assessments to follow up on psychosocial health for both patients and their families [8].

The increased survivorship as well as the documented side effects of chemotherapy and radiation have placed emphasis on the concept of health related quality of life (HRQOL) in pediatric cancer patients, both during and after treatment [18, 6, 13, 16].

The HRQOL concept encompasses the dimension physical functioning, including health status and functional status, as well as the dimension psychosocial functioning, including emotional-, social and role functioning [20]. Although sometimes used interchangeably with the concept quality of life (QOL) HRQOL has been suggested to be the appropriate term for QOL dimensions expedient to the health care services [21].

HRQOL instruments for the pediatric population generally comprise patient self-reports and parent proxy-reports. The cross-informant variance that has been well documented in both adult and pediatric samples has also been shown to be present in pediatric HRQOL instruments. Typically, there is a stronger correlation in domains reflecting external behaviors, such as physical functioning, and a weaker correlation in domains reflecting internal behaviors such as emotional and social functioning as well as pain symptoms [5]. These results underline the importance of utilizing patient self-reports as a standard for measuring HRQOL in pediatric populations even though parent proxy- reports may be warranted in cases where the child itself is too young, too cognitively impaired, too fatigue or too ill to complete the questionnaires [21, 9].

The Pediatric Quality of Life Inventory (PedsQL) is a modular instrument, designed to integrate generic and disease specific measures, and includes both self- and proxy-reports [19]. The instrument originates from The Pediatric Cancer Quality of Life Inventory (PCQL) that was designed to take into account not only the biomedical end points, such as response rate and survival, but also to focus on behavioral and emotional problems in order to capture the daily health related problems that pediatric cancer patients face [18]. Today the PedsQL instrument targets not only pediatric cancer but has been modified to cover other chronic health conditions as well, and has been translated and evaluated in a number of languages [23, 22, 9, 16, 14, 15].

The increased use of patient-reported outcome measures in both clinical and research settings requires instruments with appropriate and well documented psychometric properties [4].

The overall aim of this study was to translate the Pediatric Quality of Life Inventory 3.0 Cancer Module Scales (PedsQL 3.0) to Swedish and to assess reliability and a limited validity in a sample of Swedish children diagnosed with cancer.

## Methods

### Participants

Data for the psychometric analysis was collected among patients at the Pediatric Oncology clinic at the Queen Silvia Children’s Hospital, Sahlgrenska University Hospital, Gothenburg, Sweden. The sample consisted of in total 140 families of which 94 agreed to participate, including 63 children aged 5 to 18 years and 94 parents. Inclusion criterions for the children were; current age 2–18 years, fluency in the Swedish language and a pediatric cancer diagnosis. Inclusion criterions for the parents were; fluency in the Swedish language. Children with developmental disability and/or intellectual disability were excluded, as well as children with another significant medical disease.

### Procedure

The original version of the PedsQL Cancer Module was translated into Swedish in 2012 using forward and backward translation. The Swedish version of the questionnaire was administered to 20 families at the Pediatric Oncology clinic at The Queen Silvia Children’s Hospital, and was then followed up by structured interviews in order to detect any difficulties with the Swedish translation [10]. Some minor changes were made and reported to the MAPI Institute. After this process, the MAPI Research Institute gave approval to go ahead with the psychometric analysis of the Swedish PedsQL 3.0.

The data for the psychometric analysis was collected during the period September 2012 to January 2015 at the Pediatric Oncology Clinic at The Queen Silvia Children’s Hospital, where patients and their parents were recruited during pre-scheduled outpatient visits. Written consent was signed individually by the children (8–18 years) and their parents.

One of two research nurses informed the participating families about the study and distributed the PedsQL 4.0 and PedsQL 3.0 to children and parents. The parents were also asked to fill out a form with background variables, such as age, gender and the child’s cancer diagnosis. In the age group 2–4 years, parents alone participated. In the age group 5–7 years the children filled out the questionnaires separately from the parents, with the assistance from a research nurse, who provided help with reading and clarifying the questions. The children in the age group 8–18 years filled out the questionnaires independently, with a nurse available to assist.

### Measures

#### The PedsQL 4.0

The PedsQL 4.0 measures generic quality of life and includes both self- (child) and proxy- (parent) reports [19]. The Swedish version of the instrument has been shown to have satisfactory reliability estimates as well as satisfactory internal consistency reliability, for both child- and parent-reports, with Cronbach’s alpha values exceeding 0.70 [12]. The PedsQL 4.0 consists of 23 items (21 items in the age group 2–4 years old), covered by four scales: (1) physical functioning (eight items), (2) emotional functioning (five items), (3) social functioning (five items), and (4) school functioning (five items, three items in the age group 2–4 years).

The PedsQL 4.0 utilize a five-point Likert scale (ranging from 0 = never to 4 = almost always) for all versions, except for the child -report version 5–7 years where a 3-point Likert scale (where 0 = not at all, 2 = sometimes, 4 = a lot) is used together with a visual aid (happy face, neutral face and sad face). The items are reverse-scored and linearly transformed to a 0–100 scale (0 = 100, 1 = 75, 2 = 50, 3 = 25, 4 = 0), where higher scores indicate better HRQOL [14, 15].

#### The PedsQL 3.0

The PedsQL 3.0 was designed to evaluate disease specific HRQOL in children with cancer aged 2–18 years and includes both self- (child) and proxy- (parent) reports. Varni [20] reported satisfactory psychometric properties in a sample of pediatric cancer patients and their parents with average α-values of 0.72 for child -reports and 0.87 for parent reports for most scales. The sample consisted of all diagnostic groups and included newly diagnosed patients, recurrent patients as well as patients who were long term of treatment. The PedsQL 3.0 consists of 27 items covered by five scales: (1) pain and hurt (two items), (2) nausea (five items), (3) procedural anxiety (three items), (4) treatment anxiety (three items), (5) worry (three items), (6) cognitive problems (five items), (7) perceived physical appearance (three items) and (8) communication (three). The PedsQL 3.0 utilize a five-point Likert scale (ranging from 0 = never to 4 = almost always) for all versions, except for the child report version 5–7 years where a 3-point Likert scale (where 0 = not at all, 2 = sometimes, 4 = a lot) is used together with a visual aid (happy face, neutral face and sad face). The items are reverse-scored and linearly transformed to a 0–100 scale (0 = 100, 1 = 75, 2 = 50, 3 = 25, 4 = 0), where higher scores indicate better HRQOL.

### Statistical methods

SPSS version 22 for Windows was used for statistical analysis. Descriptive statistics were calculated for the background variables gender, age and cancer diagnosis. Internal consistency of the PedsQL 3.0 was assessed with Cronbach’s α for both child- and parent-reports in order to ensure that the items in the scale measure the same underlying concept [3]. Construct validity was determined by using the convergent validation approach, using the PedsQL 4.0 as the criterion variable. Finally, a paired samples t-test was used to test for differences between child- and parent-reports of cancer-specific HROQL.

### Ethics approval and consent to participate

The study has been approved by the Regional Ethical Review Board in Gothenburg (DNR: 004–10).

## Results

### Descriptives

Of the 140 families 94 agreed to participate in the study. Altogether 47 mothers and 23 fathers filled out the forms separately and 24 filled them out together. Only one parent-report was filled out per family. There were 51 girls and 39 boys and four who did not specify gender in the child sample (Table 1). The average age was 9.85 years old (SD = 5.4). The youngest child in the sample was one year old at the onset of cancer and the oldest was 17 years old (two nonresponses). Of the responding children 41 were diagnosed with leukemia (44%), 12 were diagnosed with a brain tumor (13%) and 41 were diagnosed with solid tumors (44%). Nine of the participating children had experienced a relapse. Fifty-one percent of the children had ongoing chemotherapy at the time of the study.

**Table 1:** Descriptives

		Diagnosis			Gender		Total
		Leukemia	Brain tumor	Solid tumour	Girl	Boy	
**Age group**	2-4 years	18	4	5	15	12	27
	5-7 years	7	1	4	10	2	12
	8-12 years	9	2	9	9	9	20
	13-18 years	7	5	23	17	16	35

Module Scales: parent proxy-report and child self -report

### Internal consistency

All cancer module scales reached or exceeded satisfactory reliability estimates of Cronbach’s alpha values (0.7) for both self- and proxy reports, except for the Communication scale where the proxy-report reached a somewhat lower estimate (0.67). The Total Scale Scores for self- and proxy-reports both reached high α-values, 0.91 and 0.94 respectively (Table 2).

**Table 2:** Alpha values for PedsQL 3.0 Cancer Module Scales; parent proxy-report and child self-report

Cancer Module	Parents (*N*=82-93)	Children (*N*=59-65)
Pain and hurt	0.86	0.84
Nausea	0.92	0.82
Procedural anxiety	0.91	0.87
Treatment anxiety	0.95	0.87
Worry	0.90	0.71
Cognitive problems	0.87	0.87
Physical appearance	0.77	0.72
Communication	0.90	0.67
Total Scale Score	0.94	0.91

Note: The average Cronbach’s α is above 0.70 for both instruments

Significant intercorrelations were found for most of the scales for both self- and proxy reports with some exceptions, as presented in Table 3.

**Table 3:** Intercorrelations between scales in PedsQL 3.0: Patient Self-Report above the Diagonal, Parent Proxy-Report below the Diagonal, Patient/Parent Correlations on the Diagonal

Cancer Module Scales	Pain and hurt	Nausea	Procedural anxiety	Treatment anxiety	Worry	Cognitive problems	Physical appearence	Communication	Total Scale Score
Pain and hurt	0.61**	0,51**	0.03	0.26*	0.41**	0.45**	0.12	0.28*	0.60**
Nausea	0.56**	0.63**	0.02	0.45**	0.52**	0.29*	0.19	0.33**	0.70**
Procedural anxiety	0.30**	0.21	0.51**	0.36**	0.22	0.36**	0.29*	0.05	0.46**
Treatment anxiety	0.38**	0.43**	0.62**	0.32**	0.31*	0.20	0.40**	0.23	0.62**
Worry	0.47**	0.60**	0.16	0.52**	0.48**	0.42**	0.37**	0.33**	0.72**
Cognitive problems	0.27*	0.29**	0.15	0.25*	0.46**	0.45**	0.32**	0.26*	0.71**
Physical appearance	0.26*	0.32**	0.14	0.38**	0.61**	0.46**	0.45**	0.23	0.59**
Communication	0.17	0.38**	0.12	0.28**	0.08	0.20	0.12	0.40**	0.51**
Total Scale Score	0.63**	0.80**	0.52**	0.72**	0.74**	0.60**	0.60**	0.49**	0.63**

*p < 0.05

**p < 0.01

Acceptable Cronbach’s alpha values were found for all age groups in the child-reports (0.67 to 0.84) (Table 4). The α-values for the parent proxy -reports were high (0.84–0.9) except for the age group 2–4 years, where Cronbach’s alpha did not reach satisfactory estimates for eight out of nine subscales (Table 5).

**Table 4:** Cronbach´s Alpha values for PedsQL 3.0 Cancer Module Scales child self-report per age group

Cancer Module	Children 5-7 yrs (*N*=12)	Children 8-12 yrs (*N*=19)	Children 13-18 yrs (*N*=33)
Pain and hurt	0.71	0.84	0.81
Nausea	0.67	0.81	0.81
Procedural anxiety	0.72	0.83	0.84
Treatment anxiety	0.71	0.81	0.80
Worry	0.72	0.80	0.78
Cognitive problems	0.74	0.81	0.80
Physical appearance	0.78	0.80	0.82
Communication	0.75	0.78	0.83
Total Scale Score	0.67	0.77	0.77

**Table 5:** Cronbach´s Alpha values for PedsQL 3.0 Cancer Module Scales parent proxy-report per age group

Cancer Module	Parents 2-4 yrs (*N*=24)	Parents 5-7 yrs (*N*=12)	Parents 8-12 yrs (*N*=19)	Parents 13-18 yrs (*N*=30)
Pain and hurt	0.55	0.87	0.87	0.87
Nausea	0.53	0.87	0.86	0.86
Procedural anxiety	0.61	0.89	0.90	0.87
Treatment anxiety	0.59	0.87	0.87	0.85
Worry	0.58	0.86	0.87	0.84
Cognitive problems	0.58	0.87	0.88	0.87
Physical appearance	0.64	0.89	0.87	0.86
Communication	0.74	0.87	0.87	0.88
Total Scale Score	0.51	0.85	0.85	0.84

### Construct validity

The PedsQL 4.0 and the PedsQL 3.0 (Total Scale Scores) were highly correlated for both proxy- (*r* = 0.71, R2 = 50%) and self-reports (*r *= 0.75, R2 = 56%). As shown in Table 6, all self- and proxy report scales of the PedsQL 3.0 showed significant correlations with the Total Scale Score of the PedsQL 4.0, except for self-report Procedural anxiety. The strongest relationships were with Pain and hurt (*r* = 0.70, 0.62 *p* = 0.01), Nausea (*r* = 0.67, 0.60, *p* = 0.01), and Cognitive problems (*r* = 0.65, *r* = 0.55, *p* = 0.01), indicating that these symptoms strongly relate to the overall HRQOL as assessed by the PedsQL 4.0.

**Table 6:** Correlations between the scales in PedsQL Cancer module and PedsQL 4.0 Generic Core Scales (Total Scale): Self and Proxy

Cancer Module Scales	Generic Core Scales (Total Scale Score)	
	Parents (***N***=88-93)	Children (***N***=108-103)
Pain and Hurt	0.62**	0.70**
Nausea	0.60**	0.67**
Procedural Anxiety	0.27**	0.09
Treatment Anxiety	0.39**	0.39**
Worry	0.54**	0.51**
Cognitive Problems	0.55**	0.65**
Physical appearance	0.48**	0.30*
Communication	0.25*	0.39**
Total cancer scale	0.71**	0.75**

*p < 0.05

**p < 0.01

### Agreement between self- and proxy-reports of PedsQL 3.0

A paired samples t-test was conducted for each scale of the PedsQL 3.0 Cancer Module to evaluate differences in child- and proxy-reports on cancer-specific HRQOL (Table 7). Overall the children reported a higher HRQOL compared to the parents (M = 77.0, 69.3; SD = 16.0, 19.7 respectively; t = 3.5; *p* < 0.01) with statistically significant differences on the scales Pain and hurt (M = 77.9, 70.3; SD = 23.8, 28.9 respectively; t = 2.5; *p* < 0.05), Nausea (M = 72.0, 59.7; SD = 25.4, 34.1 respectively; t = 3.4; *p* < 0.01), Procedural anxiety (M = 78.2, 69.7; SD = 25.7, 29.2 respectively; t = 2.5; *p* < 0.05) and Treatment anxiety (M = 89.5, 80.3; SD = 18.8, 24.9 respectively; t = 2.8; *p *< 0.01). No statistical difference was found on the scales Worry, Cognitive problems, Physical appearance and Communication. Furthermore, as shown in Table 3 there was a positive significant correlation between child- and proxy-reports for all scales in the PedsQL 3.0.

**Table 7:** Results from dependent t-test (paired sample): Differences between parents and children in how they estimate children’s HRQOL

	**Children (*N*= 59-65)**		**Parents (*N*= 82-93)**		
**PedsQL 3.0**					
**Cancer Module scales**	**Mean**	**Standard Deviation**	**Mean**	**Standard Deviation**	**t**
Pain and hurt	77.9	23.8	70.3	28.9	2.5*
Nausea	72.0	25.4	59.7	34.1	3.4**
Procedural anxiety	78.2	25.7	69.7	29.2	2.5*
Treatment anxiety	89.5	18.8	80.3	24.9	2.8**
Worry	75.6	23.8	69.2	27.6	1.9
Cognitive problems	75.3	21.3	70.2	22.0	1.7
Physical appearance	73.2	27.6	67.6	22.0	1.6
Communication	82.4	19.4	77.6	23.7	1.6
Total Scale Score	77.0	16.0	69.3	19.7	3.5**

*p < 0.05

**p < 0.01

## Discussion

This study examined the psychometric properties of the Swedish version of the PedsQL 3.0, Cancer Module. It was found that the Total Scale Score exceeded the criteria for satisfactory internal consistency, with Cronbach’s alpha values ranging from 0.91 to 0.94 for self- and proxy reports. Similarly, most of the subscales reached satisfactory estimates with Cronbach’s α values at or above 0.70. These results are in line with previous reports on the psychometric properties of disease-specific modules of the PedsQL [14–16, 9, 19] and confirm the reliability of the Swedish version of the Cancer Module Scale. When looking at the child- and parent -reports in each age-group most of the Cronbach’s α values are still satisfactory, with the exception of the values in the parent reports in the age group 2–4 years, which are below acceptable alpha values.

The PedsQL 3.0 and the generic PedsQL 4.0 total scale scores were strongly correlated for both child- and parent-reports, indicating construct validity and suggesting that cancer related difficulties were strongly related to general HRQOL. Furthermore, all but one subscale, Procedural anxiety, showed significant positive correlations with the generic PedsQL 4.0 total score scale, indicating that these scales independently relate to general HRQOL as assessed by the PedsQL 4.0.

Consistent with other studies in the field the present study found a medium to strong positive correlation between child- and parent-reports for all subscales, strengthening the validity of the PedsQL Cancer module [16, 9, 20]. The strongest correlations were found for the scales Nausea and Pain and hurt (> 0.60) and the weakest correlation was found for the scale Treatment anxiety (0.32). Except for Pain and hurt, which is typically seen as an internal problem, the intercorrelations reflect previously documented tendencies that external behaviors i.e. physical functioning, predict stronger correlations between child- and proxy-reports compared to more internal behaviors [5].

Parents reported more overall cancer-specific problems than the children themselves. Specifically, this was true for the scales Pain and hurt, Nausea, Procedural anxiety and Treatment anxiety, whereas no significant differences were found for the scales Worry, Cognitive problems, Physical appearance and communication. This is in line with previous studies on the difference between child- and parent-reports on HROQL, regardless of disease-group. One possible explanation to this is that the parents themselves have low HROQL due to the stress of having a child with a chronic disease and that this affects how they report the child’s well-being (Ingerski et al., [7]). The parents also carry the burden of responsibility and thus may be more aware of the seriousness of the situation and can foresee different scenarios and consequences, compared to especially younger children who tend to have a more “here and now” mentality. The results show that parent-proxy reports on cancer-specific HROQL may be a valuable tool in cases where the child itself (5–18 years old) for some reason is not able to answer the questionnaires, although it is important to be aware of the possible tendencies described above. In addition, the PedsQL 3.0 child- and parent -reports may serve as valuable tools in clinical practice as a means of addressing and improving the communication between children and parents when it comes to cancer-specific problems.

Some studies have also suggested that the PedsQL Cognitive problems scale can be used as a tool for identifying children in need for more extensive cognitive testing [11, 2]. Buratti et al. [2] found strong associations between 15-year old’s reports on the subscale Cognitive problems and their Full Scale Intelligence Quotient (FSIQ) (as measured using the Wechsler Intelligence Scales) as well as moderate to strong associations between the Cognitive problems parent proxy reports and the child’s FSIQ for all ages. Considering the growing body of evidence of the need for neurocognitive evaluations of patients with pediatric cancers affecting the central nervous system this may be an additional area of use for the PedsQL 3.0 and thus also a warranted focus for further studies [1].

### Limitations

The current study had some limitations. First, the study did not present data on test-retest reliability, primarily due to the challenge of being in a clinical context within a large catchment area where patients may have to travel a long way to the hospital setting. This raises ethical considerations of summoning patients and their parents to visits intended solely for test administration. Secondly, even though the overall results indicate satisfactory psychometric properties for all age groups, the parent -reports for the age group 2–4 years did not reach satisfactory alpha values, which needs to be considered when interpreting the results.

Furthermore, there was a lack of demographic data on the parents. Even though the main objective of the study was to analyze psychometric properties, which ought not to be affected by demographics, factors such as family size, age, income and occupation would have been valuable information to include in order to ensure an unbiased sample. Finally, the overall sample size in the current study was not suitable for exploratory factor analysis, therefore this would be a welcome focus for future studies [17]. Another important contribution to further strengthen the psychometric properties of the Swedish version of the PedsQL 3.0 would be to conduct a study with a comparison control group in order to measure discriminant validity.

## Conclusions

To summarize, we can conclude that PedsQL 3.0 Cancer Module Scales can be utilized as a valuable tool for measuring specific HRQOL in children with cancer in both research and clinical practice.

## Data Availability

Access to the data is restricted by the Swedish Authorities (Public Access to Information and Secrecy Act; http://www.government.se/information-material/2009/09/public-access-to-information-and-secrecy-act/). However, data can be made available for researchers after review that includes approval of the research project by both the Ethics Committee and the authorities’ data safety committees.

## Abbreviations

HRQOL: Health Related Quality of Life
PedsQL: The Pediatric Quality of Life Inventory
FSIQ: Full Scale Intelligence Quotient
